# Dentistry

**Published:** 1876-05

**Authors:** 


### DENTISTRY.

In reference to the proper preservation of the teeth, J. W. Clowes, of *Abingdon Square*, offers some useful suggestions in Life Illustrated, No. 131. He claims that legitimate dentistry is a science; but that not more than one dentist in twenty brings any credit to his profession, because there are more theorists than practitioners. We desire to assure the candid Doctor, that his is not the only calling which groans and languishes under the incubus of "theorizers." And when he claims that "ignorance and laziness are two afflictions, which weigh heavily on Dental Progress," we can give him the comforting assurance that such afflictions are not peculiar to his favorite science. We are glad that Dr. Clowes speaks in such decisive terms against the literally "*dreadful*" practice of "killing the nerve." He says, with great truth, that the nerve is the life of the tooth, and that to destroy it, is the inevitable destruction of the tooth itself. We were in hopes that, all fearless of professional prejudice, he was going to abnegate the use of the "file,"—how we instinctively scringe at the idea of the harsh grating of that villanous instrument across our ivories?—but he calls it "blessed!" Only think of a "blessed file!" Now, if it were a "file" of "Hall's Journal of Health," there would be a literal "blessing" attending its proper use. But, Dr. Clowes may be right, after all, that the "file," in Dentistry, is a "*blessing*," in knowing hands—a curse, otherwise.

